# Lycopene is superior to moringa in improving fertility markers in diet-induced obesity male rats

**DOI:** 10.1016/j.sjbs.2021.02.034

**Published:** 2021-02-18

**Authors:** Sahar M. Greish, Ghada S. Abdel Kader, Eman Z. Abdelaziz, Dalia A. Eltamany, Hanaa S. Sallam, Noha M. Abogresha

**Affiliations:** a Physiology Department, Faculty of Medicine, Suez Canal University, Ismailia, Egypt; b Human Anatomy and Embryology Department, Faculty of Medicine, Suez Canal University, Ismailia, Egypt; c Pharmacology Department, Faculty of Medicine, Suez Canal University, Ismailia, Egypt; d Nutrition and Food Science, Home Economic Department, Faculty of Education, Suez Canal University, Ismailia, Egypt; e Medical Science Department, School of Oral and Dental Medicine, Badr University in Cairo, Cairo, Egypt; f Endocrinology Division, Internal Medicine Department, University of Texas Medical Branch, Galveston, TX, USA

**Keywords:** Obesity, Moringa, Lycopene, Fertility, Oxidative stress, RC, regular chow, HFD, high-fat diet, LY, lycopene, MO, moringa, TC, total cholesterol, HDL, high-density lipoprotein, LDL, low-density lipoprotein, VLDL, very low-density lipoprotein, FSH, follicle-stimulating hormone, LH, Luteinizing hormone, PBS, phosphate buffered saline, NE, Eosin-Nigrosin, H&E, hematoxylin and eosin, iNOS, inducible nitric oxide synthase, IHC, immunohistochemistry, MDA, malondialdehyde, GSH, reduced glutathione, SOD, superoxide dismutase, ROS, reactive oxygen species, HMG-Co-A, β-Hydroxy β-methylglutaryl-CoA

## Abstract

Obesity is a condition of chronic tissue inflammation and oxidative stress that poses as a risk factor for male infertility. Moringa oleifera oil extract is known to have cholesterol-lowering properties and a potential to treat obesity, while lycopene is a potent antioxidant. We hypothesize that Moringa or lycopene may improve male fertility markers in an animal model of diet-induced obesity. Male Albino rats (n = 60) were randomized to receive regular chow (RC) or high-fat diet (HFD) for 12 weeks (n = 30 each). Animals in each arm were further randomized to receive gavage treatment with corn oil (vehicle), lycopene (10 mg/kg), or Moringa (400 mg/kg) for four weeks starting on week 9 (n = 10 each). Animals were sacrificed at 12 weeks, and blood was collected to assess lipid profile, serum testosterone, and gonadotropin levels. The testes and epididymides were removed for sperm analysis, oxidative stress and inflammatory markers, and histopathological assessment. In comparison to their RC littermates, animals on HFD showed an increase in body weights, serum lipids, testosterone and gonadotrophin levels, testicular oxidative stress and inflammatory markers, as well as sperm abnormalities and disrupted testicular histology. Moringa or lycopene reduced body weight, improved oxidative stress, and male fertility markers in HFD-fed animals with lycopene exhibiting better anti-antioxidant and anti-lipidemic effects. Lycopene is superior to Moringa in improving male fertility parameters, possibly by attenuating oxidative stress.

## Highlights

• Obesity contributes to male infertility.
• Can lycopene or moringa improve male infertility?
• Tested in a rodent model of diet-induced obesity.
• Lycopene was superior to Moringa in improving male fertility parameters.

## 1 Introduction

The global prevalence of metabolic diseases, particularly obesity, has dramatically increased worldwide (Potabattula et al., 2019). Western diet (i.e., high caloric and high saturated fat diet) and the sedentary lifestyles are predisposing factors for obesity (World Health organization, 2020). Obesity negatively affects male reproductive capacity (Davidson et al., 2015, Deshpande et al., 2019). A worldwide progressive reduction in sperm counts occurred since the mid-1900s coinciding with an increase in global obesity rates, suggesting a possible correlation (Crean and Senior, 2019).

Moringa oleifera is a species of the moringaceae family. It has various nutritional values and medicinal uses of most parts of the plant (Kumar et al., 2010). Moringa oil extract contains antioxidants (vitamin C, flavonoids, and phenolics) (Nahar et al., 2016). It possesses antimicrobial, antidiabetic, cholesterol-lowering, and liver protection properties (Caceres et al., 1992, Ghasi et al., 2000); however, its effect on obesity-induced male infertility has not been investigated.

Lycopene is a red carotenoid, with a potent antioxidant property, naturally found in red-colored fruits and vegetables like tomatoes and watermelons (Agrawal, 2018, Pourahmadi et al., 2015). Some studies have related the high consumption of lycopene with decreased risk of metabolic disorders. Researchers suggest that lycopene has beneficial health effects that could fight obesity-related pathologies (Fenni et al., 2017).

In a recent study, Moringa or lycopene was found to have comparable anti-obesity potential (Kilany et al., 2020). Moringa or lycopene was also reported to improve male fertility parameters in normal or cadmium-induced toxicity rodent model (Agrawal, 2018, Gupta and Kumar, 2002, Khalifa et al., 2016, Obembe and Raji, 2018). Yet, the effects of either product on obesity-induced male infertility remain elusive. In the current study, we aimed to assess the effectiveness of Moringa or lycopene on male fertility markers in an animal model of diet-induced obesity.

## 2 Materials and methods

### 2.1 Animals

Adult male Albino Wistar rats (age 10–12 weeks − 120–150 gm) were purchased from the Ophthalmology Research Institute in Giza, Egypt. Animals were housed in polyethylene cages (n = 5/cage) in a 12-h light–dark cycle at controlled temperature and humidity ranges of 20–27 ͦ C and 40%-60%, respectively. They were fed with a standard chow diet and water *ad libitum*. Animals were acclimatized for one week before the start of the study. All procedures and experimental protocols were reviewed and approved by the Faculty of Medicine's ethics committee, Suez Canal University.

### 2.2 Experimental procedure

Animals (n = 60) were randomized to receive regular chow (RC – Control group “C”) or high-fat diet (HFD) for 12 weeks (n = 30 each). HFD contained 20 g of fat/100 g of diet (19 g of butter oil and 1 g of soybean oil to provide essential fatty acids) and provided 19.34 kJ/g of diet, including 7.74 kJ/g as fat (Woods et al., 2003). Animals in each arm were further randomized to receive gavage treatment with either lycopene “LY” (10 mg/kg - NOW FOODS Co., USA obtained as capsules) for the C/LY and HFD/LY groups (Agrawal, 2018), or Moringa oleifera oil extract “MO” (400 mg/kg – Grenera Nutrients Private Limited, India) for the C/MO and HFD/MO groups (n = 10 each) (Bais et al., 2014). Treatment lasted for four weeks, starting on week 9. Animals were weighed weekly, then sacrificed at 12 weeks under an overdose of anesthesia. Blood samples were withdrawn from the abdominal aorta to assess lipid profile, testosterone, and gonadotropin levels. The testes were removed and immediately weighed. The caudal end of the epididymis was cut for sperm analysis. One of the testes was washed with washing buffer and then homogenized to estimate antioxidant activities. The other testis was preserved for histological and immunohistochemical analysis in a 10% formalin solution.

### 2.3 Lipid profile

Total cholesterol (TC) and High-density lipoprotein (HDL) were determined, according to Stein et al. (1986). Low-density lipoprotein (LDL) was calculated, according to Friedewald et al. (1972). Triglyceride (TG) was measured in serum, according to Wahelfed (Nagele et al., 1984). Very low-density lipoprotein (VLDL) was calculated. All kits were purchased from Biodiagnostic, Egypt.

### 2.4 Hormonal assay

Serum testosterone, follicle-stimulating hormone (FSH), and luteinizing hormone (LH) were assessed by ELISA standard kits (Biocheck, Inc. Foster City CA, USA). The procedure described in the hormone assay kits was used according to Tietz's principle (Tietz, 1995).

### 2.5 Sperm analysis

Epididymides were minced aseptically into sterile Petri dishes containing 1 ml of pre-warmed (35 °C) Dulbecco's phosphate buffered saline (PBS) and filtered through 80 μM pore size nylon-mesh. The filtrate was used for the evaluation of sperm parameters. The filtrate samples were diluted with 5 ml of pre-warmed (35 °C) Dulbecco's PBS, and the spermatozoa were counted by hemocytometer using the improved (Deep1/10 mm LABART, Germany) chamber as described (Pant and Srivastava, 2003).

Normal spermatozoa were estimated by differential staining technique using Eosin-Nigrosin stain (NE). These slides were also used for assessing the abnormal sperm morphology based on observed abnormalities of head, neck, mid-piece, and tail region of the spermatozoa. A total of 1000 spermatozoa were counted in a slide. The stained and partially stained spermatozoa were considered as dead. However, for sperm abnormalities analysis, one thousand spermatozoa (heads only or intact sperm) per animal were evaluated for head/or flagellar defects by microscopy using 100×. The head and tail abnormalities were counted per 1000 sperm count.

### 2.6 Histopathological assessment

Testicular tissues were cut and fixed in 10% formalin saline. After the fixation, the tissues were washed and processed by standard histology procedures and embedded in paraffin. Tissue sections were stained with hematoxylin and eosin (H&E). Testicular tissue was double-blindly evaluated and scored in about 50 seminiferous tubules using light microscopy at power 400 (Holstein and Davidoff, 2003).

### 2.7 Measurement of inflammatory markers in testicular tissues

Sections of 3 mm of testicular tissue were deparaffinized and rehydrated. Hydrogen peroxide was used for blocking endogenous peroxidase activity. Incubation with 5% horse serum was done for blocking non-specific antigen-binding sites. Incubation with inducible nitric oxide synthase (iNOS) antibodies (Thermo Sci, Fremont, CA, USA, 1:50) overnight at 4 °C in a humidified chamber, sections washed twice with PBS then incubated with avidin biotin peroxidase at room temperature for one hour. The sections were then washed three times with PBS, counterstained, dehydrated, and mounted. About 20 images for each group were analyzed using the immunohistochemistry (IHC) profiler plugin in the Image J program through 3,3′-Diaminobenzidine (DAB)-stained cytoplasmic option (Varghese et al., 2014). Images were acquired through an Olympus camera and microscope. Images were interfaced with IBM desktop and capture × 10 power.

### 2.8 Measurement of oxidative stress biomarkers in testicular tissues

Oxidative stress markers, namely catalase activity, lipid peroxidase, malondialdehyde (MDA), reduced glutathione (GSH), and superoxide dismutase (SOD), were measured in testicular tissues. Tissue samples were homogenized in four volumes of ice-cold Tris-HCl buffer (50 mM, pH 7.4) on a homogenizer (Ultra Turrax IKA T18 Basic, USA) for 2 min at 5,000*g* at 4 °C. SOD activity was assayed spectrophotometrically by inhibition of epinephrine autoxidation (Misra and Fridovich, 1972). MDA activity was assayed spectrophotometrically (Ohkawa et al., 1979). The Catalase activity was quantified as previously described (Aebi, 1984). GSH levels were measured according to the method described by Beutler et al. (1963).

## 3 Data analysis

Data were expressed as mean ± SEM and analyzed using the Statistical Package of Social Sciences (SPSS program, version 20, SPSS Inc., Chicago, IL, USA). The difference of mean values among groups was assessed using one-way analysis of variance (ANOVA) followed by Bonferroni's multiple comparison test. All p values reported are two-tailed, and *p* < 0.05 was considered statistically significant.

## 4 Results

Treatment with either Moringa or lycopene had no effects on RC-fed animals (C groups). The changes reported below were only observed in the HFD-fed animals.

### 4.1 Bodyweight and testicular weight

**Fig. 1:**
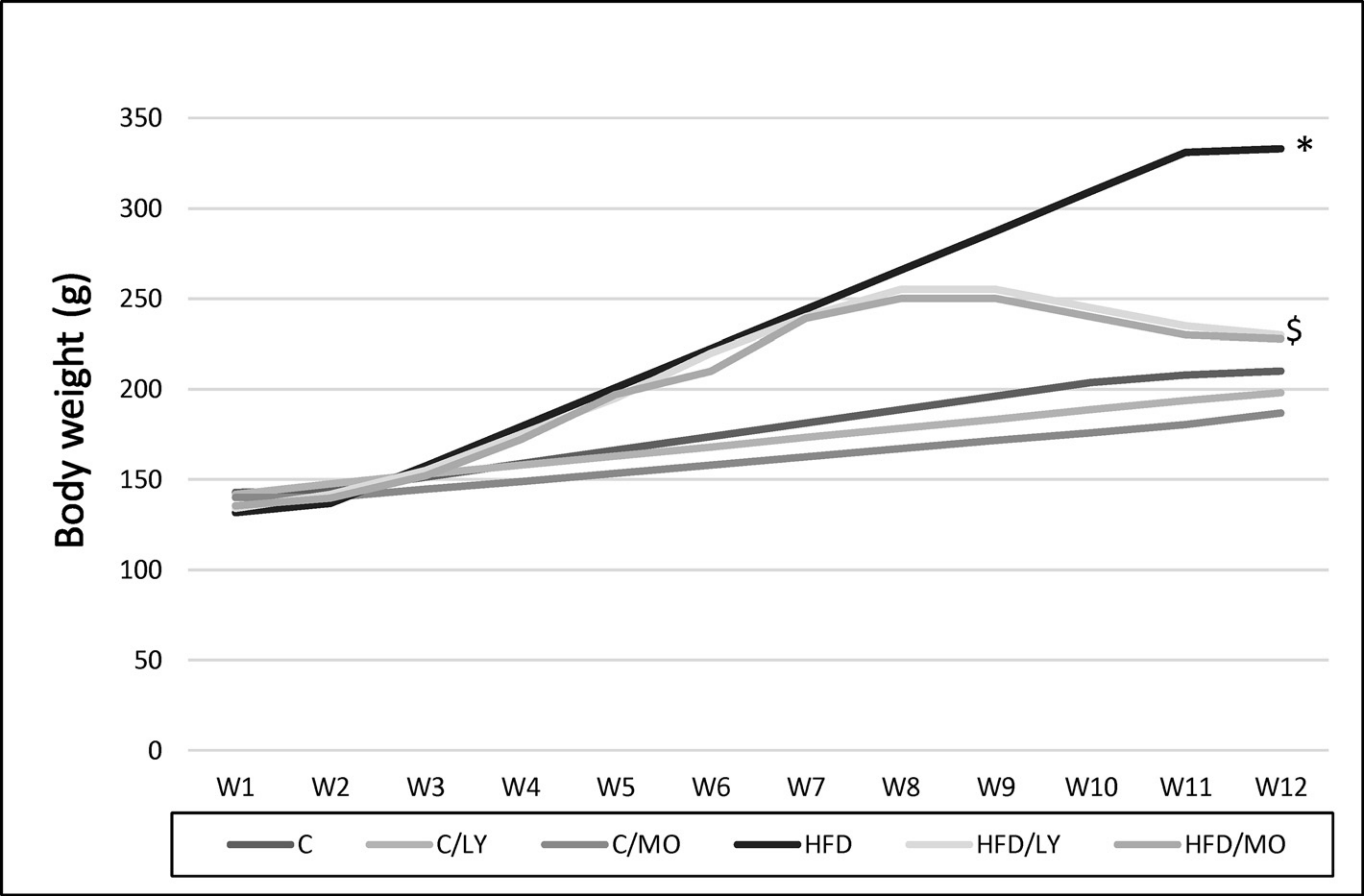
Bodyweight change over time. HFD increased all animals' body weight in the first eight weeks of the study compared to RC (*p < 0.05 each vs. HFD). Lycopene or Moringa decreased bodyweight comparably in animals on HFD (^$^*p* < 0.05 each vs. HFD). All data are expressed as mean ± SEM and analyzed using one-way ANOVA. *Significant in comparison to C group. ^$^Significant in comparison to HFD. C; control group, C/LY; control/Lycopene group, C/MO; control/Moringa group, HFD; high-fat diet group, HFD/LY; high-fat diet/Lycopene group, HFD/MO; high-fat diet/Moringa group.

Fig. 1 shows the changes in animal body weights over time in response to diet and treatment. HFD increased all animals' body weight in the first eight weeks of the study compared to RC (*p < 0.05 each vs. HFD). Lycopene or Moringa decreased bodyweight comparably in animals on HFD. Body weights at 12 weeks (g) were 230 ± 22, 228 ± 21 for lycopene and Moringa, respectively vs. 333 ± 25 for HFD (^$^*p* < 0.05 each vs. HFD). There was no significant difference between the testicular weights of the control or the treated groups (data not shown).

### 4.2 Lipid profile

Table 1 shows the values of the lipid profile. HFD increased serum levels of TC, TG, LDL, and VLDL and decreased HDL compared to RC (*p < 0.05 each vs. HFD). Treatment with either Moringa or lycopene decreased TC, VLDL, TG, and LDL, and increased HDL (^$^*p* < 0.05 each vs. HFD) with lycopene being more effective in improving the latter three (^#^*p* < 0.05 each vs. lycopene – Table 1).

**Table 1:** Lipid profile.

	TC	TG	HDL	LDL	VLDL
RC	217.2 ± 1.8	132.89 ± 4.6	45.3 ± 3.1	127.6 ± 5.2	27.07 ± 1.6
C/LY	218.2 ± 2.1	141.4 ± 4.4	47.6 ± 1.6	142.8 ± 5.9	27.7 ± 0.7
C/MO	217.2 ± 4.3	125.3 ± 4.1	48.9 ± 2.8	148.7 ± 4.5	22.1 ± 2.1
HFD	249.5 ± 0.8*	181.1 ± 1.9*	33.8 ± 202*	171.3 ± 5.4*	39.6 ± 2.1*
HFD/LY	220.5 ± 3.5^$^	139.8 ± 4.7^$^	48.7 ± 2.3^$^	139.49 ± 2.6^$^	30.15 ± 0.8^$^
HFD/MO	218.7 ± 3.7^$^	149.3 ± 4.8^$#^	40.1 ± 1.3^$#^	152.8 ± 3.6^$#^	28.7 ± 1.3^$^

### 4.3 Hormonal assay

**Fig. 2:**
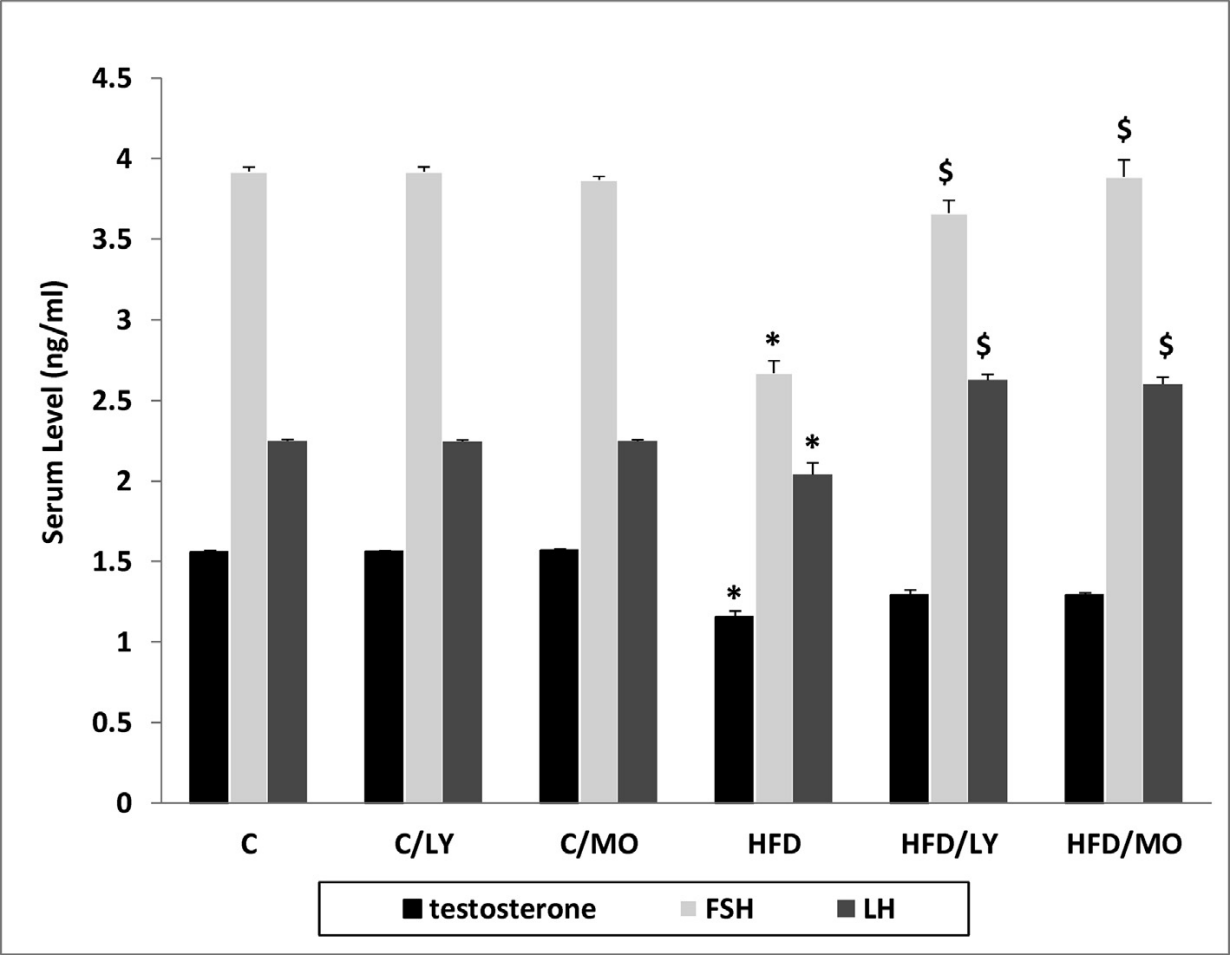
Hormonal Assay. HFD decreased serum testosterone, FSH, and LH levels compared to RC (*p < 0.05 each vs. HFD). Treatment with either Moringa or lycopene increased FSH, and LH levels comparably (^$^*p* < 0.05 each vs. HFD)**.** There was no significant difference between the changes seen in gonadotrophins in response to either treatment. All data are expressed as mean ± SEM and analyzed using one-way ANOVA. *Significant in comparison to C group. ^$^Significant in comparison to HFD. ^#^Significant in comparison to HFD/LY group. C; control group, C/LY; control/Lycopene group, C/MO; control/Moringa group, HFD; high-fat diet group, HFD/LY; high-fat diet/Lycopene group, HFD/MO; high-fat diet/Moringa group, FSH; Follicle-stimulating hormone, LH; Luteinizing hormone.

HFD decreased serum testosterone, FSH, and LH levels compared to RC (*p < 0.05 each vs. HFD). Treatment with either Moringa or lycopene increased FSH, and LH levels comparably (^$^*p* < 0.05 each vs. HFD **-** Fig. 2). FSH (ng/mL) was 3.66 ± 0.07, 3.88 ± 0.10 for lycopene and Moringa, respectively vs. 2.67 ± 0.07 for HFD (^$^*p* < 0.05 each vs. HFD). LH (ng/mL) was 2.62 ± 0.03, 2.60 ± 0.04 for lycopene and Moringa, respectively vs. 2.04 ± 0.07 for HFD (^$^*p* < 0.05 each vs. HFD). There was no significant difference between the changes seen in gonadotrophins in response to either treatment.

### 4.4 Sperm analysis

Table 2 shows the Holstein testicular score and Sperm analysis. HFD decreased all sperm analysis variables in comparison to RC (*p < 0.05 each vs. HFD). Treatment with Moringa or lycopene improved the total sperm count, sperms with normal morphology, sperms with abnormal morphology, and sperms with abnormal heads (^$^*p* < 0.05 each vs. HFD). Either treatment had no effects on the count of sperms with abnormal tail or sperms with abnormal head and tail. There was no significant difference between the changes seen in sperm analysis in response to either treatment.

**Table 2:** Holstein testicular score and Sperm analysis.

	Holstein testicular score	Total sperm count (10^6^/ml)	Sperms with normal morphology	Sperms with abnormal morphology	Sperms with abnormal head	Sperms with abnormal tail	Sperms with abnormal head & tail
C	9.1 ± 0.08	5.4 ± 0.01	892.5 ± 3.05	107.5 ± 3.05	52 ± 1.98	34.7 ± 1.61	20.7 ± 0.71
C/LY	9 ± 0.09	5.2 ± 0.02	885.5 ± 4.06	107.2 ± 2.21	51.5 ± 0.86	34.7 ± 0.71	21 ± 1.76
C/MO	8.8 ± 0.07	5.3 ± 0.02	870.2 ± 3.92	129.7 ± 3.92	56 ± 2.12	40.5 ± 1.42	32.2 ± 1.19
HFD	2.8 ± 0.01*	4.9 ± 0.05*	824.5 ± 2.73*	150.2 ± 1.24*	60 ± 0.09*	41.2 ± 0.71*	39 ± 0.90*
HFD/LY	8.3 ± 0.07^$^	5.1 ± 0.01^$^	869.2 ± 4.19^$^	130.5 ± 2.16^$^*	55.7 ± 0.62^$^	38 ± 1.02	36.7 ± 0.71
HFD/MO	8.6 ± 0.08^$^	5.2 ± 0.01^$^	873.2 ± 2.43^$^	132.5 ± 1.11^$^*	52.7 ± 1.14^$^	40.2 ± 1.19	38.2 ± 1.09

### 4.5 Histopathological assessment

**Fig. 3:**
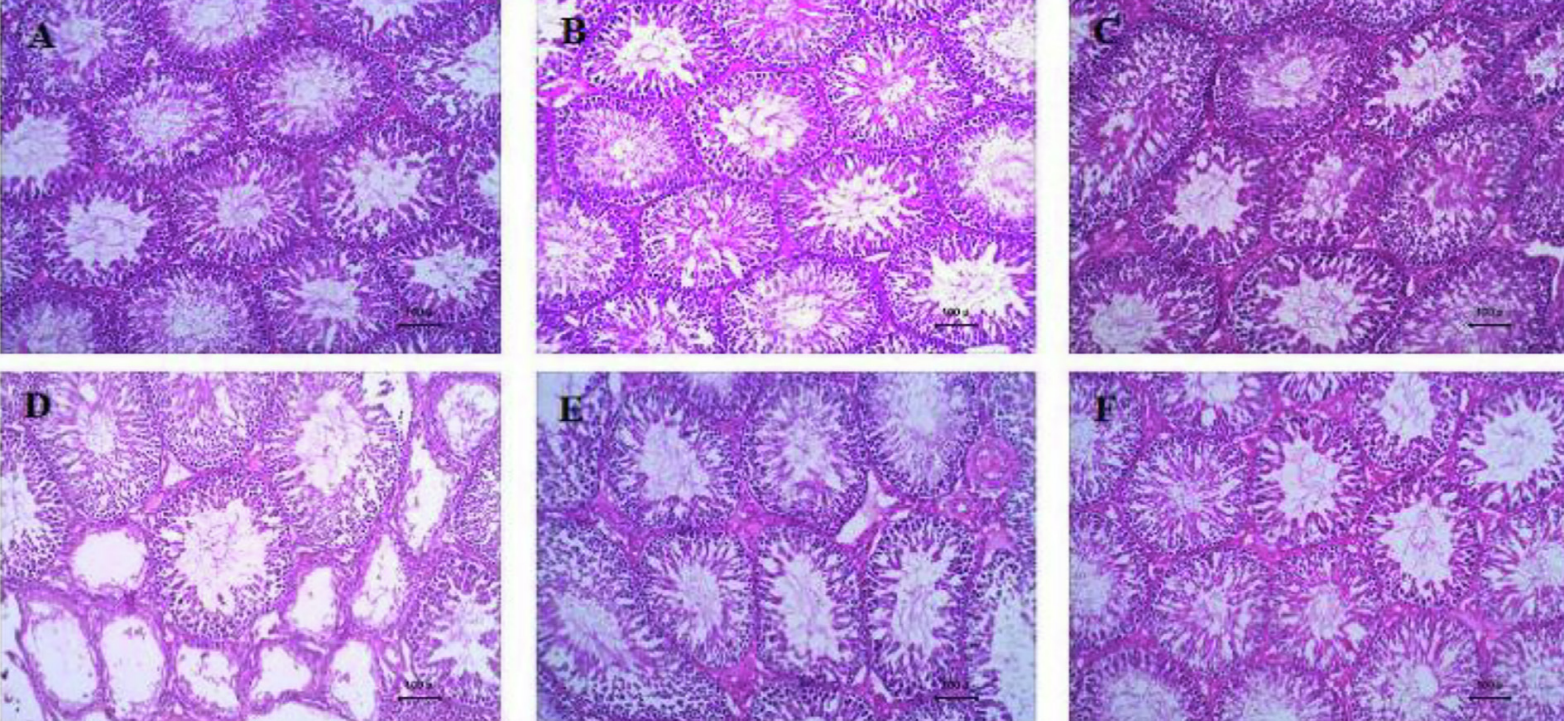
Photomicrograph of the testes in response to treatment. A: C; control group, B: C/LY; control/Lycopene group, D: C/MO; control/Moringa group, E: HFD; high-fat diet group, F: HFD/LY; high-fat diet/Lycopene group, G: HFD/MO; high-fat diet/Moringa group. (H&E × 100).

The histological structure of testicular tissue of animals in all RC-fed groups showed a regular arrangement of the seminiferous tubules presenting different stages of the spermatogenic cells starting with spermatogonia lying on the basal lamina up to sperms in the center of the tubules. In between each group of spermatogenic cells lie the Sertoli supporting cells, all being surrounded by a basal lamina. Among seminiferous tubules, Leydig cells lie within the interstitial tissue spaces **(**Fig. 3**A, B, and C)**.

In contrast, the histological structure of testicular tissue of animals in the HFD group showed several atrophic, distorted tubules with irregular basal lamina, and disrupted interstitial tissue spaces with loss of Leydig cells. We observed multiple apparently healthy seminiferous tubules with fewer spermatogenic cells, sperms, and increased spaces and vacuolations between spermatogenic cells **(**Fig. 3**D)**. Treatment with either Moringa or lycopene restored the normal architecture of the seminiferous tubules and spermatogenic cells with slight thickening in the blood vessels' tubular basement membrane and walls **(**Fig. 3**E, F)**.

Holstein score reflected the histological testicular changes observed in response to HFD compared to RC. HFD decreased Holstein scores in comparison to RC (2.8 ± 0.01 vs. 9.1 ± 0.08; *p = 0.01). Treatment with either Moringa or lycopene comparably increased Holstein scores (8.3 ± 0.07 and 8.6 ± 0.08 for Moringa and lycopene; respectively– ^$^*p* < 0.05 vs. HFD). There was no significant difference between the changes seen in sperm analysis in response to either treatment (Table 2).

### 4.6 Measurement of inflammatory markers in testicular tissues

**Fig. 4:**
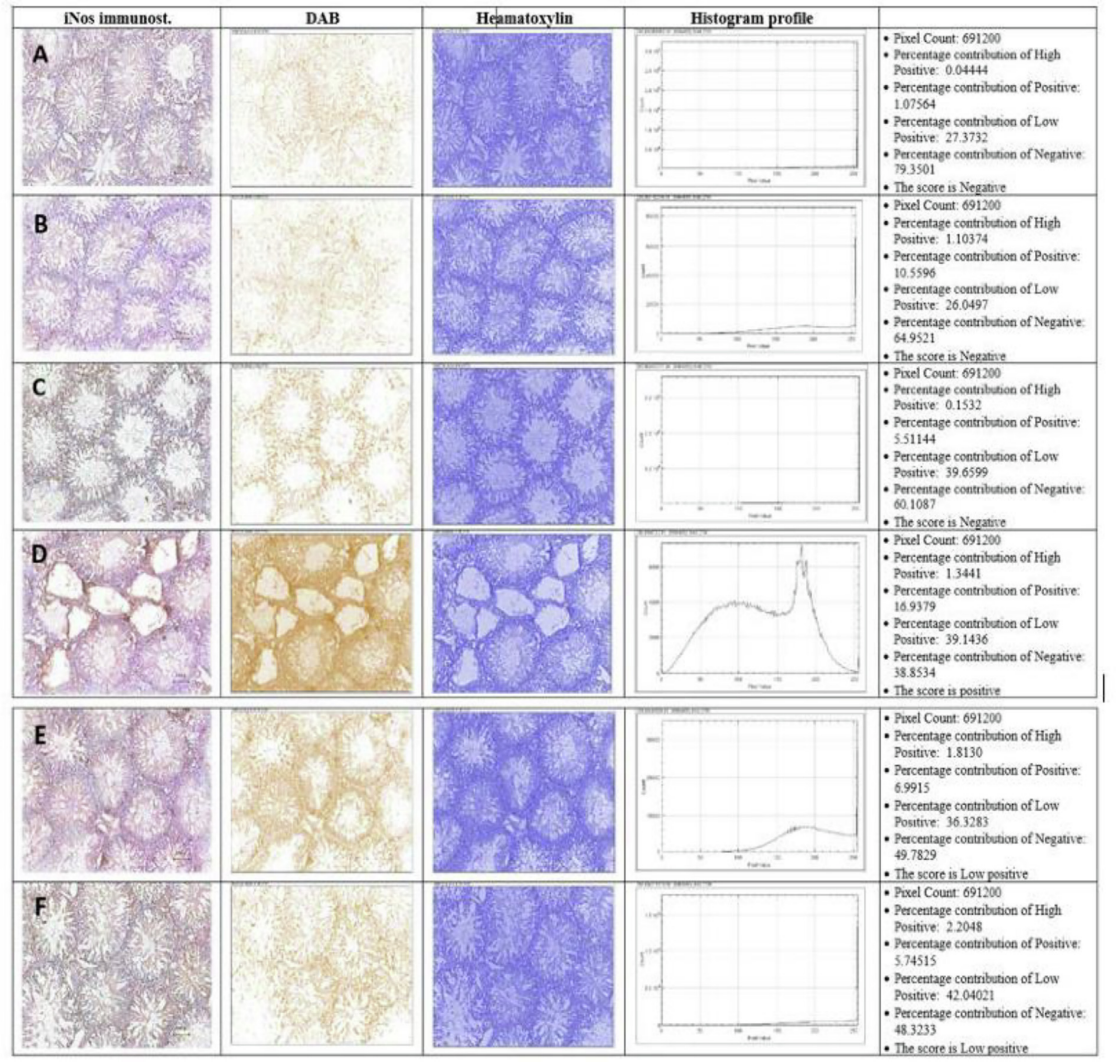
Immunostaining of the testes (using immunohistochemistry ‘IHC’ Profiler) in response to treatment. iNOS immunostaining, intensity of 3,3′-Diaminobenzidine (DAB), and hematoxylin (H) stains with each group's corresponding histogram profile and different pixels percentages of each pixel intensity (×100). A: C; control group, B: C/LY; control/Lycopene group, D: C/MO; control/Moringa group, E: HFD; high-fat diet group, F: HFD/LY; high-fat diet/Lycopene group, G: HFD/MO; high-fat diet/Moringa group.

RC decreased iNOS expression in Sertoli & germ cells (in the seminiferous tubules) and in Leydig cells (in the inter‐tubular areas - Fig. 4**A, B, and C**; respectively). In contrast, HFD increased iNOS expression (Fig. 4**D**). Treatment with either Moringa or lycopene markedly decreased iNOS expression (Fig. 4**E and F**; respectively).

### 4.7 Measurement of oxidative stress markers in testicular tissues

**Fig. 5:**
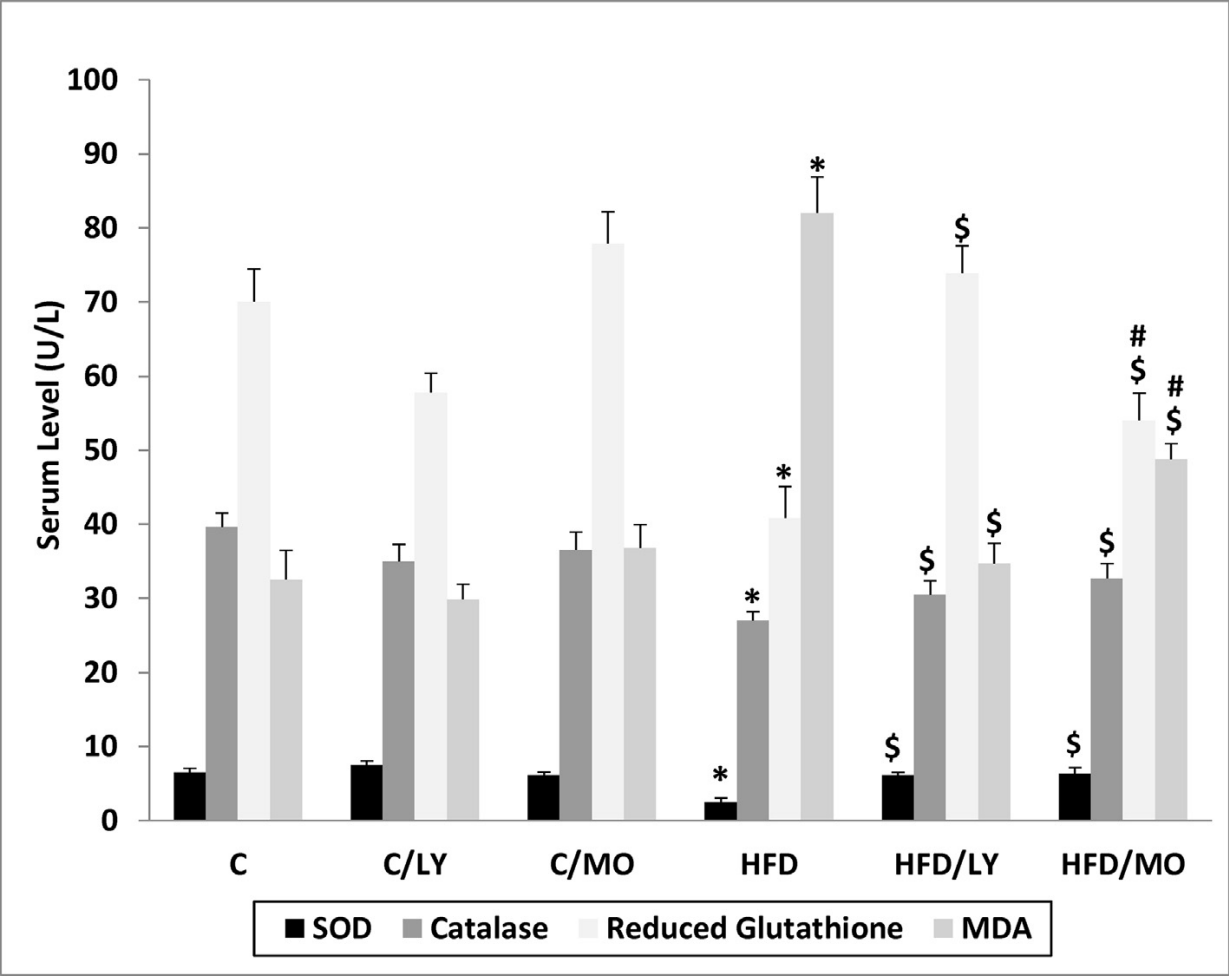
Oxidative stress marker levels in testicular tissue. HFD reduced the SOD, catalase activity, and GSH and increased MDA concentrations compared to RC (*p < 0.05 each vs. HFD). Moringa or lycopene increased SOD, catalase activity, and GSH and decreased MDA concentrations (^$^*p* < 0.05 each vs. HFD) with lycopene being more effective on the latter two (^#^*p* < 0.05 each vs. lycopene). All data are expressed as mean ± SEM and analyzed using one-way ANOVA. *Significant in comparison to C group. ^$^Significant in comparison to HFD. ^#^Significant in comparison to HFD/LY group. C; control group, C/LY; control/Lycopene group, C/MO; control/Moringa group, HFD; high-fat diet group, HFD/LY; high-fat diet/Lycopene group, HFD/MO; high-fat diet/Moringa group.

Fig. 5 shows the effects of treatments on oxidative stress markers. HFD reduced the SOD, catalase activity, and GSH and increased MDA concentrations compared to RC (*p < 0.05 each vs. HFD). Moringa or lycopene increased SOD, catalase activity, and GSH and decreased MDA concentrations (^$^*p* < 0.05 each vs. HFD) with lycopene being more effective on the latter two (^#^*p* < 0.05 each vs. lycopene). SOD (U/L) was 6.12 ± 0.85, 6.37 ± 1.70 for lycopene and Moringa, respectively vs. 2.5 ± 1.29 for HFD (^$^*p* < 0.05 each vs. HFD). Catalase activity (U/L) was 30.44 ± 4.56, 32.69 ± 4.77 for lycopene and Moringa, respectively vs. 25.99 ± 2.88 for HFD (^$^*p* < 0.05 each vs. HFD). GSH (U/L) was 73.87 ± 23.26, 54.02 ± 8.79 for lycopene and Moringa, respectively vs. 40.58 ± 10.16 for HFD (^$^*p* < 0.05 each vs. HFD - ^#^*p* < 0.05 vs. lycopene). MDA (U/L) was 34.68 ± 16.08, 48.75 ± 12.31 for lycopene and Moringa, respectively vs. 82.02 ± 18.82 for HFD (^$^*p* < 0.05 each vs. HFD - ^#^*p* < 0.05 vs. lycopene).

## 5 Discussion

This comprehensive work compared the effects of Moringa oleifera oil extract or lycopene on metabolic and fertility markers in a male rat model of diet-induced obesity. We found that: (1) In comparison to RC, HFD increased body weight, serum lipids, sperm abnormalities, serum testosterone, and gonadotropin levels, testicular oxidative stress and inflammatory markers, and disrupted testicular histology, (2) treatment with either Moringa or lycopene improved all studied parameters with lycopene exhibiting superior anti-antioxidant, and anti-lipidemic effects.

Under physiological conditions, reactive oxygen species (ROS) levels are maintained by an antioxidant system. This system is based on small scavenging molecules like GSH, and enzymes, which catalytically modify ROS into less harmful forms (Bais and Sharma, 2014). In the current work, HFD decreased SOD, catalase activity, and GSH and increased MDA concentrations compared to RC, indicating a disruption of the antioxidant system with increased lipid peroxidation, as previously reported (Kilany el al., 2020). This is conceivable as elevated oxidative stress has been implicated in obesity, a state of chronic inflammation (Fernandez-Sanchez et al., 2011).

Obesity-induced oxidative stress has been implicated in decreased Sertoli cell function, direct sperm DNA damage, and an overall reduction in sperm count (Bellastella et al., 2019; Pearce et al., 2019). Indeed, we have observed that HFD decreased gonadotropins and testosterone, FSH, and LH levels and exerted deleterious effects on testicular histopathology and sperm analysis. This is consistent with other clinical reports in obese males (Davidson et al., 2015, Sedaghat et al., 2019). Obesity has a negative impact on the systematic and regional environment pivotal for spermatogenesis and sperm maturation, which results in low sperm quality (Crean and Senior, 2019). Several mechanisms have been proposed to explain the effects of obesity on sperm functions and male fertility. These include: (1) increased conversion of androgen into estrogen in excess adipose tissue inducing hormonal imbalance, (2) increased scrotal adiposity inducing gonadal heat and suppressing spermatogenesis, and (3) increased oxidative stress and ROS. Specifically, the increased load of ROS was reported not only to induce chemical and structural changes damaging sperm nuclear structure and DNA integrity but also to disrupt seminal fluidity, which causes reduced sperm motility (Nahar et al., 2016, Pearce et al., 2019).

Inducible nitric oxide synthase is not expressed under normal circumstances but is upregulated during inflammatory conditions. We observed iNOS overexpression in Sertoli and Leydig cells in animals on HFD, which was in accordance with other studies (Sedaghat et al, 2019). Such overexpression reflects the increased testicular inflammatory burden (Zaitone et al, 2015) and possible inhibition of steroidogenesis (O'Bryan et al., 2000).

Medicinal plants have been used for treating obesity for millennia. Moringa or lycopene reduced the weight of HFD-fed animals significantly, suggesting an anti-obesity potential of either (Bais et al., 2014; Pierine et al., 2014, Xie et al., 2018; (Kilany et al., 2020). Either treatment exerted anti-lipidemic effects on HFD-fed animals, consistent with other reports (Fenni et al., 2017, Pierine et al., 2014, Jiang et al., 2015; (Kilany et al., 2020) with lycopene being more effective. Researchers showed that Moringa or lycopene depressed the activity of β-Hydroxy β-methylglutaryl-CoA (HMG-Co-A) reductase enzyme, the rate-regulating enzyme for cholesterol biosynthesis (Khan et al., 2017, Mozos et al., 2018). In addition, others linked the salutary effects of Moringa on body weight and dyslipidemia to improving visceral fat adipokine gene expressions (Soleymaninejad et al., 2017).

Either treatment had antioxidant effects on HFD-fed animals, consistent with other reports (Mozos et al., 2018, Jiang et al., 2015), yet lycopene showed better results. Indeed, researchers showed that lycopene reduces oxidative stress by scavenging free radicals neutralizing ROS, and preventing the damage of lipids, proteins, and DNA (Agrawal, 2018, Khan et al., 2017, Metwally et al., 2017).

Importantly, either treatment improved testosterone and gonadotropin levels, selective semen analysis parameters, and restored the testicular structure of HFD-fed animals. Lycopene was shown to improve sperm count, motility, viability, and morphology, possibly attributed to its antioxidant properties (Agrawal, 2018, Gupta and Kumar, 2002). Moringa was also shown to improve spermatogenesis and preserve testicular morphology, possibly attributed to the presence of flavonoids, which can lessen oxidative stress (Khalifa et al., 2016, Obembe and Raji, 2018).

Either treatment showed a minimal intensity of iNOS immunostaining, denoting the reduction of iNOS expression in HFD-fed animals. These findings highlight Moringa and lycopene's anti-inflammatory properties, consistent with other reports (Kahn et al., 2017; Vergara-Jimenez et al., 2017). Moringa was shown to decrease the gene expression and production of inflammatory markers in Ralph and William (RAW) macrophages (Vergara-Jimenez et al., 2017). Lycopene exerted several anti-inflammatory effects besides modulating inflammatory mediators, such as inhibition of leukocyte mobilization, stabilization of mast cells, and inhibition of gene expressions involved in inflammation. Lycopene also reduced the secretion of metalloproteinases by macrophages and inhibited T lymphocyte activation (Khan et al., 2017, Vergara-Jimenez et al., 2017).

A limitation of this work is the lack of dose–response studies with various treatments. Dose-response could have provided an insight into the identification of the best treatment dose. Additionally, our diet-induced obesity model could have been more realistic if we would have replaced the HFD with a palatable, energy-dense, westernized, or cafeteria-food diet to mimic a real-life ultra-processed food diet. Further studies are warranted to test the effects of cafeteria diet on infertility parameters in rodents.

In summary, several factors contribute to lowering male fertility, including environmental, nutritional (e.g., HFD), pharmacological, and others. In a lab. controlled environment, obesity was teased out as a factor by conducting the study in a diet-induced obesity rat model. Obesity negatively affects male fertility and sperm quality through mediating testicular inflammation and oxidative stress. Lycopene or Moringa are potent natural antioxidants that can ameliorate the reprotoxicity and potentially treat obesity-induced male infertility with lycopene exhibiting superior anti-antioxidant and anti-lipidemic effects.

## Author contribution

All authors made substantial contributions to conception and design, acquisition of data, or analysis and interpretation of data; took part in drafting the article or revising it critically for important intellectual content; gave final approval of the version to be published; and agree to be accountable for all aspects of the work.

## Declaration of Competing Interest

The authors declare no conflict of interest.
